# The Function of TRIM25 in Antiviral Defense and Viral Immune Evasion

**DOI:** 10.3390/v17050735

**Published:** 2025-05-20

**Authors:** Qianxun Liu, Shantong Peng, Jiani Wei, Zhenzhen Xie

**Affiliations:** 1 School of Basic Medical Sciences, Jiangxi Medical College, Nanchang University, Nanchang 330031, China; lqx20040127@163.com (Q.L.); pst15005391360@163.com (S.P.); dlwjnini@163.com (J.W.); 2 Medical Department, Queen Mary School, Nanchang University, Nanchang 330031, China

**Keywords:** TRIM25, RIG-I, MDA5, ZAP, antiviral, immune evasion

## Abstract

Tripartite motif (TRIM) 25 is a member of the TRIM E3 ubiquitin ligase family, which plays multiple roles in anti-tumor and antiviral defenses through various pathways. Its RBCC and SPRY/PRY domains work cooperatively for its oligomerization and subsequent activation of ligase activity. TRIM25 expression is regulated by several proteins and RNAs, and it functionally participates in the post-transcriptional and translational modification of antiviral regulators, such as RIG-I, ZAP, and avSGs. Conversely, the antiviral functions of TRIM25 are inhibited by viral proteins and RNAs through their interactions, as well as by the viral infection-mediated upregulation of certain miRNAs. Here, we review the antiviral functions of TRIM25 and highlight its significance regarding innate immunity, particularly in antiviral defense and viral immune evasion.

## 1. Introduction

Ubiquitination is a crucial and fundamental process of post-translational modification (PTM) consisting of the covalent attachment of ubiquitin to protein substrates mediated by a cascade of E1 ubiquitin-activating enzymes, E2 conjugating enzymes, and E3 ligase enzymes [1]. E3 ligases, or E3 ubiquitin ligases, catalyze the direct transfer of ubiquitin to substrate proteins, leading to their proteasomal degradation. E3 ligases are classified into three major types—RING (really interesting new gene), HECT (homologous to E6AP C-terminus), and RBR (RING-between-RING)—each employing distinct catalytic mechanisms for ubiquitin transfer. Tripartite motif (TRIM) proteins constitute a subset of RING-type E3 ubiquitin ligases. TRIM proteins play essential roles in various regulatory processes, such as cell signaling, carcinogenesis, protein interaction, and DNA repair [2]. Evolving from the same ancestral gene, TRIM proteins contain a highly conserved RING–B-box–coiled-coil (RBCC) domain at their N-termini [3]. However, because of evolutionary processes, the C-terminal domains of TRIM members (CⅠ-CⅣ) exhibit distinct characteristics [3]. The C-IV subfamily of TRIM proteins contains a unique PRY/SPRY (B30.2) domain at its C-termini; it is found in many other proteins, including SplA kinase and ryanodine receptors (RyRs) [4,5]. As shown in previous research, the allosteric effects of multiple TRIM protein domains and TRIM protein oligomerization are crucial for the catalytic activity of TRIM E3 ligase [6]. Furthermore, TRIM family proteins have the ability to interact with RNAs [3]. Evidence from mRNA interactome capture studies and protein–RNA crosslinking capture experiments has revealed that a variety of TRIM proteins exhibit RNA-binding capabilities [3,7,8]. TRIM25 is a member of the C-IV subfamily and was recently found to have an RNA-binding preference and functional domain through a study employing iCLIP2 [9]. During this interaction, RNA can function as a scaffold to facilitate conformational changes in TRIM family proteins and subsequent protein–protein interactions [9,10]. Basically, TRIM proteins mediate two types of polyubiquitination: K48-linked ubiquitination for proteasomal degradation and K63-linked ubiquitination for cellular signaling and cytokine synthesis [6,11,12]. Both types of ubiquitination shed light on the possibility that TRIM25 regulates antiviral defense by modulating antiviral proteins (AVPs) and virus-related components involved in innate immunity. For instance, previous studies have identified the possible viral restriction structures in TRIM25: retinoic acid inducible gene I (RIG-I) and the zinc-finger antiviral protein (ZAP) [13,14,15]. This multifaceted domain structure allows TRIM25 to engage in diverse biological activities, making it a pivotal component in the cellular response to viral infection [6,16].

Several recent reviews have focused on the roles of TRIM25 and its underlying mechanisms in cancer and inflammatory diseases [17,18,19]. TRIM25 has been shown to play critical roles in various cell death pathways, including apoptosis, pyroptosis, necroptosis, ferroptosis, and autophagy, indicating its potential as a therapeutic target in cancer [17]. However, recent advances in our understanding of the antiviral functions of TRIM25—particularly its intricate interactions with viral RNAs and proteins, as well as its regulation of antiviral responses in innate immunity—have not been comprehensively addressed in existing reviews. Therefore, we conducted this review to synthesize studies on the TRIM25–viral component interactions that regulate antiviral defense and viral immune evasion. We will discuss the basic structure of TRIM25 and its regulatory effects on immune signaling.

## 2. General Structure and Gene Regulation of TRIM25

### 2.1. Gene Regulation of TRIM25

The TRIM25 gene is located on human chromosome 17q22, composed of 25kb of genomic DNA and nine exons [20]. Functionally, the TRIM25 gene encodes TRIM25, an inducible E3 ubiquitin ligase, which is predominantly located in the cytosol and can be redistributed into the organelles within a cell following viral infection. The TRIM25 gene is a kind of interferon-stimulated gene (ISG) whose promoter can be activated by type I interferon (IFN1α and IFNβ), and its expression is induced during viral infection [19,20]. The TRIM25 gene contains interferon-stimulated response elements (ISREs) in the first intron that can recruit signal transducers and the activator of transcription 1 (STAT1), mediating IFN-stimulated TRIM25 transcription [21]. Additionally, TRIM25 was initially identified as an estrogen-responsive finger protein (EFP), which acts in an estrogen-dependent manner based on its estrogen-responsive element (ERE) at the 3′ untranslated region (3′UTR) [19,20,21]. The binding of estrogen to estrogen receptor α (ERα) and its interaction with ERE promote the activation of the proximal promoter and TRIM25 transcription [22]. Apparently, both IFNs and estrogen can induce TRIM25 mRNA transcription and subsequent protein expression. Furthermore, TRIM25 expression is negatively regulated by a myriad of microRNAs (miRNAs) [19,23,24,25,26]. These miRNAs can bind to the 3′ untranslated region (3′ UTR) of TRIM25 mRNA, inhibiting TRIM25 protein translation and promoting TRIM25 mRNA degradation, thereby downregulating TRIM25 expression [24,25]. Several studies have shown that miR-30a and miR-202-5p bind to the 3′UTR of the TRIM25 gene, negatively regulating its expression [27,28,29]. MiR-30a is upregulated by coxsackievirus B3 (CVB3), inhibiting TRIM25 expression and subsequent RIG-I ubiquitination, which facilitates CVB3 replication and enhances viral infection [27]. This suggests that the miR-30a-mediated suppression of TRIM25 and RIG-I contributes to the evasion of IFN-I-mediated antiviral responses, promoting CVB3 infection. Similarly, miR-202-5p is induced by red-spotted grouper nervous necrosis virus (RGNNV) infection. Its overexpression inhibits TRIM25-mediated RIG-I ubiquitination, impairing RIG-I-dependent innate immune responses and promoting RGNNV infection [28]. These findings advance our understanding of viral evasion mechanisms against immune responses and have clinical relevance, including therapeutic potential with respect to targeting these regulatory pathways in viral infections and related diseases.

In addition, E3 ligases, such as TRIM25, are subjected to various post-translational modifications, such as ubiquitination [10], phosphorylation [30], ISGylation [31], and sumoylation [32]. The K117 of TRIM25 undergoes autoubiquitination [10]. Given that TRIM25 is an RNA-binding protein (RBP), the binding of TRIM25 to its own mRNA 3′UTR region can promote its autoubiquitination, limiting its expression [10]. Furthermore, 14-3-3, acting as an enhancer of TRIM25 binding and RIG-I ubiquitination [33], can promote TRIM25 autoubiquitination and inactivation in response to the BamH1 P fragment leftward open reading frame1 (BPLF1) protein of Epstein–Barr virus (EBV), reducing IFN production [34]. Whether RNA viruses can also interfere with the antiviral activity of TRIM25 by binding 14-3-3 remains to be explored. The linear ubiquitin chain assembly complex (LUBAC), a novel multiprotein E3 ligase complex, can add K48-linked ubiquitin chains to TRIM25, resulting in the formation of linear polyubiquitin chains [35,36]. Conversely, ubiquitin-specific protease 15 (USP15) can remove these LUBAC-mediated linear ubiquitin chains, thereby regulating the functions of TRIM25, including its antiviral activity [37]. Moreover, the TRIM25 protein can be post-translationally phosphorylated at the threonine 91, serine 97, serine 100, tyrosine 278, and threonine 427 residues, and TRIM25 tyrosine 278 phosphorylation is important for TRIM25-mediated RIG-I activation [30]. In response to the virus, TRIM25 in the cytosol can interact through its SPRY/PRY domain and be phosphorylated at the tyrosine 278 residue by c-Src tyrosine kinase, enhancing TRIM25-mediated RIG-I ubiquitination [30]. Notably, ubiquitination can be modulated by interferon-stimulated gene (ISG)15 and the small ubiquitin-like modifier (SUMO), which regulate protein expression and transportation [38]. Firstly, TRIM25 can undergo auto-ISGylation for self-modifications at lysine 177 between the RING and CC domains, enabling TRIM25 to transfer ISG15 to itself [31]. This modification requires the RING domain of TRIM25 and the support of the E2-conjugating enzymes UbcH6 and UbcH8 [31]. Originally, ISG15 should be transferred from E2 enzymes to 14-3-3σ, while TRIM25 ISGylation leads to the inhibition of its ISG15 E3 ligase activity toward 14-3-3σ [31,39]. Additionally, TRIM25 does not seem to directly interact with SUMO [32], but it is crucial for SUMO-mediated protein stabilization [32]. Specifically, TRIM25 can interact with SUMOylated proteins and promote their ISGylation, leading to TRIM25-dependent protein stabilization [32]. Furthermore, SUMO can promote the nuclear translocation of TRIM25 [32], in which TRIM25 may interact with RNAs and regulate various biological pathways.

Even though TRIM25 is involved in regulating gene and protein PTM, TRIM25 gene transcription is induced by type I interferon and estrogen, and its mRNA is targeted by a variety of miRNAs to inhibit its expression after transcription. Thus, TRIM25 expression is transcriptionally and post-transcriptionally regulated by various regulators and modifiers to maintain the balance of its biological functions.

### 2.2. Structural Insights into and Higher-Order Conformation of the RBCC Domain

**Figure 1:**
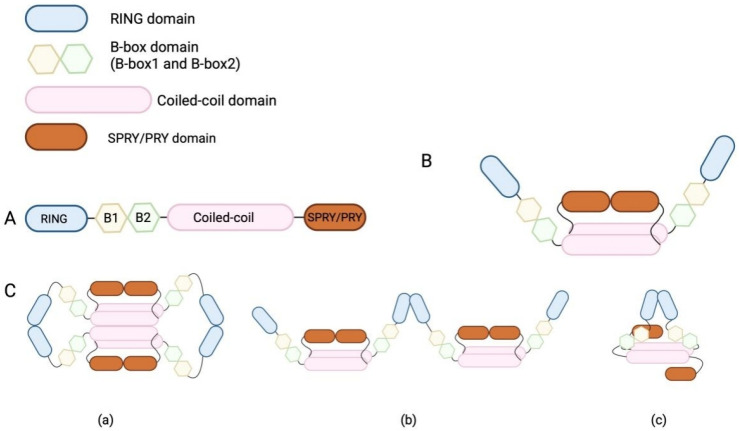
Illustration of TRIM25 monomer and dimer structures and possible RING dimerization modes. (**A**) The structure of TRIM25, possessing a RING domain, a B-box domain, a coiled-coil domain, and a SPRY/PRY domain. (**B**) The structure of a TRIM25 dimer. (**C**) The possible modes of RING dimerization. This dimerization stabilizes the closed conformation, activates thioester intermediates, and facilitates ubiquitin transfer in chain synthesis. (**Ca**) The RING domains of two TRIM25 dimers interact to form a TRIM25 tetramer. (**Cb**) TRIM25 dimers link laterally in an end-to-end configuration for the formation of TRIM25 tetramer. (**Cc**) B-box domain contributes to RING dimerization by binding to the CC domain, which shortened the distance between two RING domains within a TRIM25 dimer. (This figure was created using BioRender. Liu, Q.X. https://BioRender.com/r07m967, accessed on 7 March 2025).

As a highly conserved domain in the TRIM family, TRIM25 contains an RBCC domain that is composed of a RING domain, a B-box domain, and a coiled-coil (CC) domain (Figure 1A). The RING domain, known to be a zinc-finger protein, has a series of cysteine and histidine residues that coordinate two zinc ions, stabilizing the structure of this domain [40]. Functionally, the RING domain has the catalytic activity of an E3 ubiquitin ligase and is responsible for protein–protein interactions during viral restriction. The E3 ubiquitin ligase activity of the RING domain is necessary for the transfer of ubiquitin monomers from E2-conjugating enzymes to protein substrates [41]. This process is particularly important for the TRIM25-mediated antiviral responses in innate immunity. Through this catalytic activity, the RING domain interacts with RIG-I and ubiquitinates the CARD domain of RIG-I, whose feedback enhances the production of type I interferon. The B-box domain is one of the conserved domains in TRIM family proteins, and it is usually located between the RING and CC domains. There are two types of B-boxes, namely, B-box1 and B-box2, and each B-box domain is composed of approximately 50 amino acids [42]. Some TRIM family proteins have only a B-box2 domain, but TRIM25 has both B-box1 and B-box2 near its N-terminal sites [40,42]. As both the B-box and CC domains have a zinc-finger-binding ability similar to that of the RING domain, the B-box domain and CC domain enable themselves to bind zinc ions and maintain structural stability [42]. The B-box and CC domains have highly conserved motifs within the TRIM family, and the CC domain is typically positioned at the C-terminal of the B-box. Functionally, the CC domain facilitates the formation of homodimers or heterodimers in TRIM family proteins [40,41]. The CC domain of TRIM25 is the most important basic unit for its dimerization. The central region of the CC domain of TRIM25 contains numerous hydrophobic residues, which enable the antiparallel arrangement of the CC domain and subsequent tight binding of the helical CC domain through hydrophobic interactions [4], promoting TRIM25 dimer formation (Figure 1B). There is an L2 linker region between the CC and SPRY domains, and this short helical domain also undergoes self-association to form a dimer after the dimerization of CC helices [5]. TRIM family proteins usually form a conserved four-helix bundle, composed of a CC helix dimer and an L2 linker helix dimer. Given that individual TRIM proteins have unique sequences in the central region of the CC domain [5], these TRIM proteins have difficulty forming heterodimers [5]. However, the same branch of proteins with a similar central region sequence in the TRIM family can form heterodimers through CCD-mediated monomer interactions [5,43]. The specific and important roles of the central region of the CC domain in stimulating the dimerization of TRIM proteins have been demonstrated. In the proposed structure of the TRIM25 dimer, the RING and B-box domains are situated at the ends of the CC helical dimer, while the SPRY domain is in close proximity to the CC domain [12].

Several studies have extensively examined the functions of the RING domain, B-box domain, and CC domain in enhancing the catalytic activity of TRIM25. First, the CC domain of TRIM25 promotes the formation of a RING-based TRIM25 dimer through the RING domains to interact with E2 enzymes/ubiquitin and moves adjacent to the C-terminal alpha helix to complete the interaction [42,43]. Interestingly, one study has reported that the RING domain of TRIM25 is capable of mediating ubiquitination without requiring oligomerization for its activation [42], and CC domain-induced RING domain-based dimerization greatly enhances its catalytic capacity [42]. However, the RING monomer is insufficient for the formation of a polyubiquitin chain [44]. Considered to be an obligate dimer, the TRIM25 dimer undergoes further dimerization, which is believed to give rise to a closed conformation with E2/ubiquitin and facilitate further ubiquitin transfer [5,42,45]. Specifically, TRIM25 interacts with E2-conjugating enzymes through a conserved E2–E3 interface, while a RING dimer binds to the same ubiquitin, the proximal RING domain binds to the ubiquitin surface patch, and the distal RING domain interacts with ubiquitin, possibly through an acidic element outside the RING domain [5,42,45], thereby stabilizing a closed conformation, which activates ubiquitin thioester intermediates and further facilitates ubiquitin transfer [42]. Previous studies have shown that both the CC and B-box domains contribute to RING domain dimerization [40,41,42], while neither domain has an effect on the corresponding catalytic activity. Notably, it has been suggested that the B-box domain enhances RING dimerization by binding to the CC domain, thereby reducing the distance between the two RING domains in TRIM25 dimers [42] (Figure 1(Cc)). This hypothetical process, however, was deemed infeasible due to the inability of these two RING domains within TRIM25 dimers to achieve intramolecular linkage [5]. Consequently, alternative models for the RING domain association have been proposed, suggesting that TRIM25 may form higher-order aggregates [6]. For instance, the RING domains of two TRIM25 dimers can interact to form a tetramer (Figure 1(Ca)), or TRIM25 dimers can link laterally in an end-to-end configuration to form extended TRIM25 dimer chains [6] (Figure 1(Cb)). Therefore, the B-box and CC domains of TRIM25 are responsible for RING dimerization, and this dimerization greatly enhances catalytic activity during ubiquitin chain synthesis. The multiple domains of TRIM25, the structure of its dimer, and the possible mechanisms and working models of RING domain dimerization are illustrated in Figure 1.

### 2.3. The Structure of the SPRY/PRY Domain and Its Protein Interaction and RNA-Binding Ability

The PRY/SPRY domain is derived from the fusion of the SPRY and PRY domains [40]. It exhibits a β-sandwich structure, composed of two antiparallel β-sheets, one six-stranded sheet, and one antiparallel seven-stranded sheet [46]. Both the monomers and dimers of the PRY/SPRY domain exist at high protein concentrations, while only the monomers are present at low protein concentrations and form SPRY/PRY dimers in a concentration-dependent manner [46]. Dimerization occurs through concave contact between the C-terminal residues of one domain and the six-stranded beta-sheets of another [4,46], providing insight into PRY/SPRY’s interactions with other proteins. Specific loops and residues on the surface of the SPRY domain are crucial for recognizing and binding to target proteins. These regions can be tailored to interact with particular motifs or domains on other proteins [46]. In innate immunity, the PRY/SPRY domain of TRIM25 binds directly to the CARDs domain of RIG-I, triggering the RIG-I/IFN signaling pathway [47]. However, direct interactions between the SPRY domain and viral proteins can disrupt RIG-I ubiquitination, thus inhibiting the host immune response [19]. Additionally, the SPRY domain can interact with the M2-2 proteins of HMPV or the V proteins of paramyxovirus to inhibit the ubiquitination of RIG-I and the subsequent signaling pathway [48,49,50]. Moreover, a 39-amino-acid stretch in the C-terminal PRY/SPRY domain of TRIM25 can bind to RNA or RNA viruses to facilitate this domain’s interaction with AVPs, such as RIG-I [10,11]. Besides the PRY/SPRY domain, the L2 linker between the CC and SPRY domains contains a lysine-rich region of the 7K motif for RNA binding [12]. However, the 7K motif itself does not exist independently as an RNA-binding element. A study has shown that the 7K motif and SPRY domains are required for structural adaption in RNA binding [12]. Previous studies have revealed that the 7K motif in TRIM25 is critical for RNA virus infection-induced IFN production because its mutation significantly decreases the RNA-binding capacity of TRIM25 [10,12]. However, the binding of WT TRIM25 to RNA significantly enhances TRIM25-mediated RIG-I ubiquitination and IFN production [12]. Although the interaction between TRIM25 and RIG-I and the activation of downstream signaling pathways do not necessarily involve the binding of TRIM25 to the same RNA [12], this RNA-binding process is critical for the E3 ligase activity of TRIM25.

## 3. Biological Functions of TRIM25

### 3.1. Basic Functions of TRIM25

TRIM25 can directly or indirectly regulate various basic biological processes, such as cell proliferation, the cell cycle, protein stabilization, and mRNA translocation. These basic biological processes are critical in cancer development, inflammation, and innate immunity, and most of them are related to the E3 ligase activity and RNA-binding ability of TRIM25.

**Figure 2:**
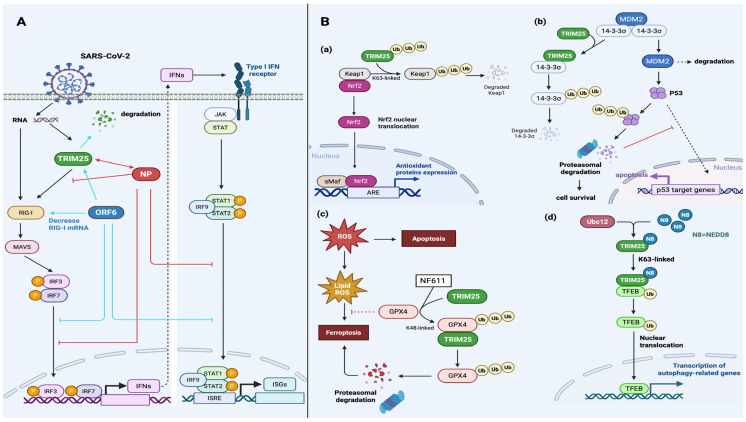
Biological functions of TRIM25 in SARS-CoV-2 infection and cancer cell survival. (**A**) SARS-CoV-2 proteins participate in viral immune evasion by interfering with antiviral signaling. SARS-CoV-2 viral RNA can activate TRIM25 and RIG-I for the subsequent aggregation of MAVS, which regulate IRF3/7 and IFN production. The IFNs produced bind to a type I IFN receptor to activate the JAK/STAT signaling pathway, leading to the production of ISGs. Both the SARS-CoV-2 nucleocapsid protein (NP) and ORF6 proteins influence the nuclear translocation of IRF3/7 and STAT. The NP recruits G3BP2 and TRIM25, while ORF6 mediates TRIM25 degradation, limiting TRIM25-mediated RIG-I ubiquitination. (**B**) TRIM25-mediated ubiquitination regulates multiple biological pathways. (**Ba**) TRIM25 can lead to the degradation of Keap1, resulting in the nuclear translocation of Nrf2 and the production of antioxidant proteins. (**Bb**) 14-3-3σ can be degraded by TRIM25-mediated ubiquitination, which contributes to the degradation of Mdm2 and p53. (**Bc**) The inhibitory effect of GPX4 on ferroptosis is negated by the participation of TRIM25 in the treatment of N6F11. (**Bd**) Neddylated TRIM25 can induce the nuclear translocation of TEFB, which is responsible for the transcription of autophagy-related genes. (This figure was created using BioRender. Liu, Q.X. https://BioRender.com/zi5oxce, accessed on 7 May 2025).

Recent studies have revealed novel mechanisms by which TRIM25 regulates cancer cell growth and migration based on its catalytic activity [20,22,51]. TRIM25 can modify protein targets through K63- or K48-linked polyubiquitination [11]. K63-linked polyubiquitination can activate multiple signaling pathways, promoting protein localization or modulating protein–protein interaction [22]. In contrast, K48-linked polyubiquitination can mediate protein degradation [11]. Interestingly, upregulated TRIM25 expression is usually detected in many types of tumor cells, especially in hormone-responsive tumors, such as breast cancer, prostate cancer, ovarian cancer, and endometrial cancer, because of its estrogen-responsive characteristics [18]. Upregulated TRIM25 expression is also detected in lung cancer, gastric cancer, and hepatocellular carcinoma [19]. TRIM25 recognizes 14-3-3σ and promotes its degradation to reduce its activity in breast and endometrial cancer [17,19,50], promoting cancer cell survival and proliferation. In particular, the degradation of 14-3-3σ induced by TRIM25 limits Mdm2 autoubiquitination and degradation, thereby destabilizing p53, attenuating p53-dependent tumor-suppressive signaling [17] (Figure 2(Bb)). Additionally, TRIM25 can target Keap1, a regulatory protein of Nrf2, and the proteasomal degradation of Keap1 increases the nuclear localization of Nrf2 to initiate anti-oxidative responsive gene expression [52] (Figure 2(Ba)). Furthermore, TRIM25 enhances tumor cell survival, as treatment with N6F11 to target TRIM25-mediated GPX4 degradation selectively induces cancer cell ferroptosis [17,53,54,55] (Figure 2(Bc)). Moreover, TRIM25 can target the phosphatase and tensin homolog (PTEN) by mediating its K48- and K63-linked polyubiquitination to enhance PI3K/AKT signaling and cancer cell growth [17,56,57]. Interestingly, TRIM25 can mediate breast cancer cell autophagy via UBC12-enhanced TRIM25 neddylation, which increases transcription factor EB (TFEB) K63-linked polyubiquitination and nuclear translocation and thus activates autophagy-related gene expression, leading to drug resistance [17,58] (Figure 2(Bd)). The specific mechanisms are shown in Figure 2B.

Notably, the role the binding of TRIM25 to mRNAs plays has been described in regard to both cancer therapy and antiviral responses. A recent study has shown that TRIM25 can bind to caspase-7 mRNA to attenuate caspase-7 expression by reducing its mRNA stability in colon cancer cells [59]. Furthermore, TRIM25 can bind to the 5′ UTR of caspase-2 mRNA to inhibit its translation, but this binding does not interfere with the overall structure and integrity of caspase-2 mRNA [60]. Interestingly, TRIM25 expression is negatively targeted by miRNAs, which bind to the 3′ UTR of TRIM25 mRNA to inhibit its translation and promote its degradation [24,25]. In acute myeloid leukemia (AML), TRIM25 can promote tumor cell proliferation and migration, and its expression is negatively regulated by miRNA-137 [23]. Similarly, miR-365 can also inhibit the expression of TRIM25 in NSCLC [61].

The ubiquitination activity of TRIM25 enhances tumor cell survival and modifies regulatory proteins in innate immunity, e.g., the activation of RIG-I and TRAF proteins [62,63,64]. Based on its transcriptional and post-transcriptional regulatory mechanisms, TRIM25 serves as an oncogenic factor promoting the proliferation and migration of cancer cells. Furthermore, TRIM25 can bind viral RNAs to regulate the defense against viral infection in innate immunity.

### 3.2. TRIM25 Is Targeted to Inhibit RIG-I Signaling

SARS-CoV-2 is a type of single-stranded positive-sense RNA virus [65]. The 5’ end of its viral genome has reading frames of 1a and 1b encoding 16 nonstructural proteins (NSP1–16), while the 3’ end of the viral genome contains multiple subgenomic RNAs for the expression of four structural proteins, namely, spike (S), envelope (E), membrane (M), and nucleocapsid proteins, as well as several accessory proteins, including ORF6 [65,66]. Previous studies have reported that nucleocapsid proteins (NPs), NSPs, and many SARS-CoV-2 accessory proteins have antagonistic effects on IFN responses [65,67,68,69,70,71,72]. The detailed mechanism underlying interactions between TRIM25 and virus accessory proteins (especially SARS-CoV-2) will be discussed in the following section (Table 1).

SARS-CoV-2 infection can upregulate TRIM25 expression, which activates RIG-I and ZAP through ubiquitination and thus induces IFN production [67,82,83]. A study has reported that upregulated TRIM25 expression can limit SARS-CoV-2 replication [84], suggesting that upregulated TRIM25 expression is a viral-infection-induced compensative antiviral response. During SARS-CoV and MERS-CoV infection, the viral NP can inhibit the innate immune system and the antiviral activity of TRIM25 to promote viral invasion [62,67]. In fact, the SARS-CoV-2 NP can interact with TRIM25 at its N-terminal aa 1-360 and aa 1-175, C-terminal aa 361-419, and intermediate region aa 252-360, allowing it to interact with the SPRY and RING domains of TRIM25 to inhibit RIG-I polyubiquitination and IFN production because the direct binding of viral NPs to TRIM25 inhibits the binding of TRIM25 to RIG-I [70]. Interestingly, co-immunoprecipitation revealed that the viral NP, together with TRIM25 and RIG-I, formed a protein complex in epithelial cells [68]. The protein complex did not affect TRIM25 aggregation, but it did reduce RIG-I ubiquitination, impairing IRF activation [68]. It is likely that the viral NP mainly acts as an interposing element between TRIM25 and RIG-I, impeding their interaction to diminish IFN production [68]. In addition, the viral NP can recruit TRIM25 and G3BP2, enhancing their interactions, which, in turn, interfere with RIG-I signaling [73]. Consistently, the viral NP interacts with G3BP1 to impede the formation of stress granules (SGs) [72], wherein TRIM25 co-condensates with G3BP1 to enhance the polyubiquitination of RIG-I and other antiviral proteins [74]. The SARS-CoV-2 NSP5 and NP counteract antiviral SG formation by inhibiting virus-induced IFN production and RIG-I-mediated antiviral immunity [72]. Specifically, the viral NP dose-dependently attenuates TRIM25-mediated RIG-I antiviral signaling [68,70]. Low-dose NPs inhibit RIG-I ubiquitination by hampering the interaction between TRIM25 and RIG-I [68,70], reducing IFN-I production, while high-dose NPs enhance the phosphorylation of STAT1 and STAT2 and their nuclear translocation to induce ISG translation for antiviral defense [70].

Notably, the SARS-CoV-2 ORF6 protein can inhibit the antiviral effect of IFNs through a variety of mechanisms. Firstly, ORF6 can significantly reduce the levels of RIG-I ubiquitination by decreasing RIG-I mRNA levels by 50%, targeting TRIM25-related proteasomal degradation and inhibiting RIG-I activation and IFN production [65]. Secondly, ORF6 can directly inhibit the nuclear translocation of IRF3/7 and STAT1 for the induction of IFN and ISG activity and the triggering of the IFN-mediated signaling pathway [65].

Various types of viruses interact with TRIM25 to inhibit TRIM25-mediated RIG-I ubiquitination. For example, the NS1 protein of influenza A virus (NS1-A) can interact with the CC domain of TRIM25 to form stable complexes, preventing TRIM25-mediated RIG-I ubiquitination [76,77], or directly interact with RIG-I to inactivate IRF-3 and other transcription factors, inhibiting IFN transcription [85]. In contrast, through its RBD domain, the NS1 protein of influenza B virus (NS1-B) interacts with TRIM25, but not with RIG-I, to block the inhibition of the NS1-B CTD region upon RIG-I ubiquitination, enhancing IFN and inflammatory cytokine production in lung epithelial cells [78]. These two different effects of NS1 proteins from two types of influenza viruses on RIG-I signaling highlight the various pathogens and antiviral responses involved in early infection with influenza A and B virus.

Accordingly, the NP, NSP5, and ORF6 of SARS-CoV-2 virus interact with TRIM25 to inhibit RIG-I/IFN signaling, forming ternary complexes or degrading TRIM25. The specific signaling pathways and consequences of NP and ORF6 interference with antiviral processes are shown in Figure 2A. NS1-A and NS1-B proteins can interact with TRIM25, and both can inhibit or enhance RIG-1 ubiquitination and IFN production through various mechanisms.

### 3.3. TRIM25 Regulates Viral RNAs for Immune Evasion

A previous study has shown that TRIM25 may prefer to bind to dsRNA or ssRNA instead of DNA [12]. TRIM25 can bind to RNA through its SPRY domain and 7K motif [74]. After binding to RNA, TRIM25 can more effectively interact with RIG-I and act as a ubiquitin ligase for RIG-I ubiquitination [12]. Furthermore, PR-2B, an emerging viral clade of Dengue virus serotype 2 (DENV-2), can produce high levels of subgenomic flavivirus RNA (sfRNA), which sequence-dependently binds to TRIM25 and prevents its deubiquitination, inhibiting RIG-I-induced IFN production [81]. The PR-2B clade replaced the PR-1 clade during an epidemic lasting from 1995 to 2007 [86], and this process was accompanied by significant changes in the sequences of its 3’ UTR [86]. The increased expression of this sfRNA in viral genes is thought to promote viral replication and transmission and inhibit the action of TRIM25 [81]. The relatively low level of gRNA in this case may stimulate RIG-I and MDA5 in a moderate way, diminishing IFN production [81]. As a result, these would increase virus replication and blood viral concentrations, promoting DENV transmission between humans and mosquitoes [81].

The DENV-2 PR-2B clade can inhibit the RIG-I-dependent antiviral pathway. However, its sfRNA interacts with TRIM25 to inhibit virus replication and spread through a RIG-I-independent pathway. A previous study has shown that TRIM25, even in the case of an RNA-binding deficient TRIM25 mutant, can interact with IAV RNA, indicating that TRIM25 binds to viral RNA in a novel manner and is crucial for its antiviral activity [75]. TRIM25 binds to positive-strand IAV RNA to promote RNA instability and degradation, inhibiting IAV replication and assembly [75].

TRIM25’s RNA-binding ability is critical to its capacity to restrict viral replication and spread through RIG-I-dependent or -independent pathways. Further investigations are necessary to explore the novel RNA-binding mode of TRIM25 in the absence of currently known RBD.

## 4. Interaction Between TRIM25 and Antiviral Host Factors

### 4.1. TRIM25 Facilitates the Production of IFNs and Cytokines, Mainly Through RIG-I/MDA5 Signaling

RIG-I and melanoma differentiation-associated gene 5 (MDA5) are RIG-I-like receptors (RLRs), important components of the pattern recognition receptor (PRR), and they can be ubiquitinated to activate a series of antiviral immune responses [87]. RIG-I and MDA5 can recognize the pathogen-associated molecular patterns (PAMPs) of RNA viruses, resulting in their activation and subsequent ubiquitination, leading to changes in their structures [6,88]. Notably, RIG-I and MDA5 are structurally similar and contain a DExD/H-box helicase-like domain with ATPase and translocase abilities, a C-terminal repressor regulatory domain (CTD/RD), and two amino-terminal caspase recruitment domains (CARDS) [87]. However, they have different CTD structures, which are responsible for sensing different viral RNAs [89]. The CTDs of both RIG-I and MDA5 contain a zinc-binding domain, which possesses a positively charged groove with different conformations that recognize RNA with different preferences [62]. Consequently, RIG-I prefers to recognize short, double-stranded RNA (dsRNA) with 5′-triphosphate (5′-ppp) groups [62]. In contrast, MDA5 exhibits high affinity for the binding of long dsRNA replication intermediates and poly (I:C) without a preference for 5′ppp groups [62,90]. Specifically, RIG-I primarily senses negative-strand RNA viruses, such as Sendai virus (SeV), vesicular stomatitis virus (VSV), Newcastle disease virus (NDV), and influenza viruses, while MDA5 predominantly recognizes picornaviruses and coronaviruses [89]. After RNA recognition and binding to their DExD/H-box helicase-like domains, both RIG-I and MDA5 are activated and ubiquitinated, undergo ATP-dependent conformation changes, and form CTD-based oligomers [89].

**Figure 3:**
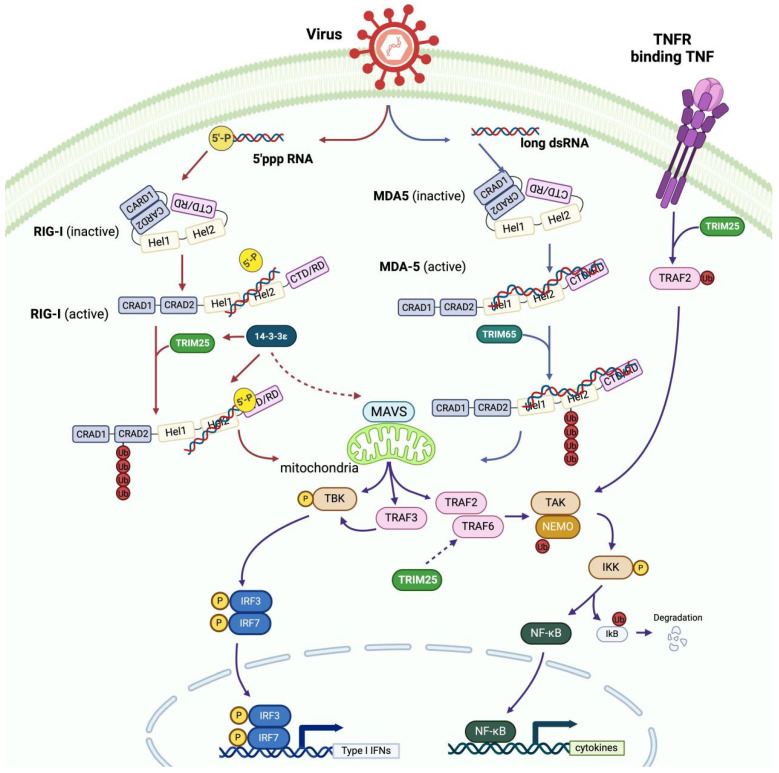
TRIM25 regulates the RIG-I- and MDA5-mediated antiviral and TRAF2/TNFα signaling pathways. RIG-I/MDA5 remains in a closed conformation until the binding of viral RNA to open the RIG-I CARDs domain. TRIM25 ubiquitinates RIG-I to trigger RIG-I/IFN signaling. TRIM25 also binds to 14-3-3ε to enable RIG-I activation and accumulation and interacts with TRAF6 to induce downstream signaling. In addition, TRIM25 can modify TRAF2, a member of the TRAF family, independently of RIG-I/MDA5 signaling. (This figure was created using BioRender. Liu, Q.X. https://BioRender.com/h64h569, accessed on 17 March 2025).

The conformational changes of RIG-I and MDA5 expose their RD-repressed CARDs and DExD/H-box helicase-like domains for further ubiquitination [89]. The activation of RIG-I and viral components induces the RING domain-based oligomerization of TRIM25, which is vital for the transfer of ubiquitin to RIG-I after the TRIM25 SPRY/PRY domain interacts with the RIG-I CARD [5,87]. Specifically, TRIM25 ubiquitinates the RIG-I tetramer at K172 [62,85], while TRIM65 ubiquitinates the MDA5 dimer or oligomer at the K743 of helicase-like domain 2 [87]. TRIM25- and TRIM65-mediated K63-linked ubiquitination enables residues of RIG-I and MDA5 to bind downstream adapter molecules called mitochondrial antiviral-signaling proteins (MAVs) on the outer membrane of mitochondria [91,92]. Although TRIM25 does not directly interact with MAVS [63], it promotes the interaction of ubiquitinated RIG-I with MAVS. Additionally, TRIM25, together with RIG-I and a member of the 14-3-3 family, forms a ternary complex that guides the translocation of RIG-I from the cytosol onto the mitochondrial cell membrane [33]. The exposure of the RIG-I CARD facilitates its binding to TRIM25 and 14-3-3ε. In the absence of infection, 14-3-3ε is mainly localized in both the cytosol and membrane, offering the possibility of the subsequent redistribution of RIG-I [33]. Studies have found that 14-3-3ε interacts with RIG-I following an acute infection and promotes the ubiquitination of RIG-I by TRIM25 to stabilize 14-3-3ε, TRIM25, and the RIG-I translocon [33]. This translocon carries the ubiquitinated RIG-I from the cytosol to the mitochondrial cell membrane [33], where RIG-I further activates MAVs on the mitochondrial outer membrane [93]. The activated MAVs form prion-like aggregations, which recruit various proteins [93]. RIG-I and MDA5 share a similar signaling pathway downstream of the MAVs, which predominantly recruit TANK-binding kinase 1 (TBK1) and TRAF proteins, such as TRAF2, TRAF3, and TRAF6 [19]. TRAF3 activates TBK1–IRF3 signaling, in which TBK phosphorylates the interferon-regulatory factors of IRF3 and IRF7 [90,93,94]. In contrast, TRAF2 and TRAF6 recruit NEMO, which further facilitates TAK1-mediated nuclear factor kappa-B (NF-kB) kinase epsilon (IKKε) complex activation [93]. Consequently, the activated IRF3/7 and NF-kB induce the expression of type I interferon and inflammatory cytokines [94], hence restricting viral replication and impeding viral spreading [83,92]. The whole process whereby TRIM25 and other regulatory proteins mediate the RIG-I/MDA5 signaling pathway is summarized in Figure 3, together with the TRIM-based mediation of TNF/TRAF2 signaling.

Interestingly, even though TRIM25 is considered to be uninvolved in RIG-I/MAV downstream signaling, it can regulate MDA5-mediated antiviral signaling by increasing the levels of TRAF6 ubiquitination [93]. However, there is no evidence demonstrating direct interaction between TRAF6 and TRIM25 [93]. Further investigations are required to uncover the molecular mechanisms by which TRIM25 regulates the MDA5-mediated NF-kB antiviral response [93]. Moreover, TRIM25 can interact with TRAF2 independent of RLR-related signaling [64]. During viral infection, TRIM25 can mediate K63-linked TRAF2 ubiquitination, which activates TNFα-induced NF-kB signaling [64].

Even though TRIM25 does not directly ubiquitinate MDA5, it can regulate the levels of TRAF6 to modulate MAV downstream signaling. Meanwhile, TRIM25 is a crucial enhancer of RIG-I because it can promote K63-linked RIG-I ubiquitination and activation, and the RIG-I, TRIM25, and 14-3-3ε translocon promotes the accumulation of RIG-I on the mitochondrial membrane. Further research is required in order to understand the molecular mechanism underlying the actions of TRIM25 in regulating MDA5-mediated antiviral defense.

### 4.2. TRIM25 Localizes in Stress Granules During Viral Infection

SGs are cytoplasmic components generated in response to stress [74]. They are aggregates of proteins and RNAs and serve as hubs for antiviral signaling [95]. As a platform for antiviral signaling, SGs can recruit and accumulate various AVPs, such as RIG-I and MDA5 [95]. Interestingly, SGs contain G3BP1/2, which can co-condensate with TRIM25 in a dsRNA-dependent manner [74]. Specifically, G3BP1 directly interacts with TRIM25 at the “404-PTFG-407” motif, which is located between the CC and PRY/SPRY domains [74]. This motif, together with its 7K motif and SPRY/PRY domain, possesses the RNA-binding ability of TRIM25 and contributes to the subsequent innate immune response [74]. SGs recruit RIG-I and TRIM25 and serve as the platform for their interaction and RIG-1 ubiquitination. Interestingly, the binding of TRIM25 to G3BP1 significantly enhances TRIM25 ubiquitination activity [74], which is a target for viral evasion. Indeed, previous studies have shown that some viral proteins can bind to both TRIM25 and G3BP2 proteins to negatively regulate RIG-I antiviral signaling [72,73,74]. The NP of SARS-CoV-2 can interact with G3BP1 to impede the formation of SGs [72,73,74] and inhibit subsequent RIG-I signaling. Additionally, this NP can also recruit TRIM25 and G3BP2 to form TRIM25–G3BP2–NP interactomes [73], which inhibit RIG-I/IFN signaling and promote SARS-CoV-2 immune evasion. Furthermore, the H1N1 NS1-A protein can inhibit TRIM25 dimerization to reduce the co-condensation of TRIM25 and G3BP1 [74].

Hence, SGs serve as a platform for many antiviral actions because they contain many regulatory proteins, including TRIM25 and G3BP1/2, and the NP of SARS-CoV-2 and the H1N1 NS1-A protein can interfere with their recruitment and thus inhibit antiviral responses during corresponding viral infections [72,73,74].

### 4.3. TRIM25 Serves as a Co-Factor for the ZAP-Mediated Antiviral Response

ZAP (also called PARP13), encoded by the ZH3HAV1 gene, is a host antiviral factor [13]. Mammalian ZAP is mainly expressed in two isoforms—namely, ZAPS (short) and ZAPL (long)—through different splicing [96]. Both ZAPS and ZAPL contain CCCH zinc-finger (ZnF) domains at their N-termini, a crucial factor for their RNA binding [97]. ZAPL, but not ZAPS, has a C-terminal poly (ADP-ribose) polymerase (PARP)-like domain, which is important for its antiviral activity but does not exhibit catalytic activity [97]. Interestingly, ZAP, like TRIM25, prefers to detect CpG-rich sequences of viral RNA [80], suggesting that both ZAP and TRIM25 may share similar antiviral functions [10].

Previous mutagenesis studies have revealed that the RNA binding of both ZAP and TRIM25 is important for their restriction of viral replication, particularly for mutation in the CpG binding site of ZAP [10,98]. Secondly, the interaction of ZAP with TRIM25 is critical for their inhibition on viral protein translation, and TRIM25 is required for ZAP-mediated antiviral responses [98,99]. To confirm the contribution of TRIM25 to ZAP RNA binding, mutants of the TRIM25 RNA-binding domain SPRY and SPRY/7K motif were constructed, and the results indicated that the loss of TRIM25 RNA-binding ability greatly reduces ZAP antiviral activity [14]. Thus, TRIM25 acts as a co-factor of ZAP and is crucial for the ZAP-mediated antiviral process [14]. ZAP has a potent ability to restrict viral mRNA expression [100]. By constructing ubiquitin mutants lacking the ability to engage in K63- or K48-linked ubiquitination, the researchers behind one experiment revealed that the K63 mutant significantly inhibited the interaction between ZAP and RNA [98], while the K48 mutant or wild type regarding ubiquitin expression enhanced target RNA interaction [98]. However, during viral infection, the ubiquitination of ZAP does not directly affect its antiviral ability. Interestingly, TRIM25-mediated K63- and K48-linked polyubiquitination and IFN induction also regulate alternative ZAP splicing because TRIM25 uses RNA as a scaffold for K48- and K63-linked polyubiquitination [10,80,98]. K63-linked polyubiquitination has a more important effect on virus binding [82,98]. Further experiments unveiled that TRIM25-mediated proteasomal degradation is crucial for the maintenance of normal levels of ZAP in the host environment [10]. The interaction between ZAP and TRIM25 may initiate the recruitment of TRIM25, which enhances the ubiquitination and activation of the cellular regulatory substrates that inhibit viral mRNA expression, particularly during JEV infection [99].

Apparently, the RNA binding of TRIM25 and ZAP is necessary for TRIM25-mediated ZAP ubiquitination, while ZAP isoforms recognize target mRNA and recruit RNAs for the degradation of target RNA, inhibiting viral replication.

The viral ribonucleoprotein complex (vRNP) of Ebola virus (EBOV) can interact with TRIM25. The vRNP is composed of viral genomic RNA, nucleoprotein (NP), and its chaperon [79], among which the NP is ubiquitinated by TRIM25, leading to the dissociation of NPs from the viral genome [79]. Interestingly, the dissociation of NPs from the viral genome triggered by TRIM25 exposes CpG-rich dinucleotides in the viral genome for ZAP recognition and antiviral defense [79,80]. Furthermore, Meyerson et al. demonstrated that TRIM25 exerts a nuclear role by acting on the vRNPs (viral RNA segments, polymerase, and viral nucleoproteins) of IAV, independent of ZAP, specifically inhibiting the viral RNA synthesis of influenza A virus [101]. This mechanism is independent of its ubiquitin ligase activity and the interferon pathway, involving the inhibition of RNA chain elongation by obstructing the movement of RNA into the polymerase complex [101]. These findings indicate that TRIM25 carries out its antiviral functions through a novel pathway, independent of RIG-I-related signaling [79].

Because of ZAP’s antiviral ability to recognize RNA with CpG-rich sequences, the genomes of several RNA and small DNA viruses affecting vertebrates can undergo gene evolution, which reduces the frequency of CpG dinucleotides after invading a host environment [80]. For example, the genomes of the β- and γ-herpesvirus families have undergone CpG suppression [80]. Another example of CpG suppression is SARS-CoV-2, which is stronger than SARS-CoV and MERS-CoV [100]. Although ZAP prefers to recognize CpG-rich sequences, ZAP, particularly ZAPL, can also recognize and restrict SARS-CoV-2 replication in a low-CpG environment [100]. Indeed, ZAPL inhibits S and NP translation more efficiently than mRNA expression in SARS-CoV-2 virus, an ability partially explained by the fact that there are twice as many CpG sequences in the gene encoding for NPs [100]. This evidence further supports the notion that ZAP prefers to recognize CpG-rich sequences and engages in important antiviral activity, even in CpG-sequence-suppressed viruses. To the best of our knowledge, SARS-CoV-2 forms RNP with its NPs and viral RNAs, while TRIM25 can sequester NPs from RNAs to expose CpG-rich sequences in the viral genome for ZAP recognition. However, whether TRIM25 and ZAP can target SARS-CoV-2 vRNPs for their antiviral processes remains to be determined.

## 5. Discussion

In this review, we have discussed the crucial role of TRIM25 in defending against viral evasion via its E3 ubiquitin ligase activity, which contributes to the RIG-I-related antiviral effect and its RNA-binding capacity to inhibit viral replication. A recent study has shown that TRIM25 can ubiquitinate RIG-I to induce the production of IFNs as well as regulate TRAF6 and IKK activities and MDA5/MAVS downstream signaling [94]. Furthermore, both TRIM25 and ZAP prefer to recognize CpG-rich sequences, which triggers TRIM25 to use RNA as a platform to modify ZAP via K48- and K63-linked ubiquitination [13,82]. Additionally, TRIM25 interacts with the NP of EBOV to expose its CpG-rich sequences to ZAP [79]. It is unknown whether TRIM25 can interact with the NPs of other viruses to enhance ZAP antiviral activity and how TRIM25 activates ZAP. It is possible that the binding of TRIM25 to viral RNA leads to TRIM25 conformational changes that enhance TRIM25-mediated ZAP ubiquitination. Apparently, the activation of antiviral proteins by TRIM25 depends on its E3 ligase activity and RNA-binding ability. Inhibiting TRIM25’s dimerization, its catalytic activity, and its interaction with RIG-I will attenuate its antiviral activity and thus promote viral immune evasion. However, TRIM25 can also directly inhibit the expression of viral RNA by degrading it [75]. Even without ubiquitinating NPs in vRNPs, TRIM25 can bind to NPs and RNA to inhibit RNA elongation, thus inhibiting viral replication and strengthening the innate immunity [101].

Thus, TRIM25 can bind to viral RNA, inhibit RNA elongation [101] and viral replication [102], and mediate RNA degradation [75], limiting viral spreading. TRIM25 serves as an enhancer of ubiquitination-mediated RIG-I activation and participates in SG formation and ZAP antiviral processes. Additionally, various viral components interact with the CC and SPRY/PRY domains of TRIM25 to inhibit its conformational changes and antiviral protein activation, facilitating viral immune evasion [62,85,94]. Furthermore, some viral proteins recruit regulatory proteins to inhibit the subsequent activity of TRIM25 [73]. Moreover, viral RNA binds to TRIM25 to suppress its deubiquitination and inhibit the activation of TRIM25 E3 ligase activity [81].

Despite advances in our understanding of the critical roles of TRIM25 in antiviral defense and viral immune evasion, limitations remain that necessitate further investigation. We propose several future directions and perspectives for research in this specific area. First, an in-depth exploration of the regulatory mechanisms governing TRIM25 expression is needed. For instance, elucidating how viral proteins and RNAs modulate TRIM25 levels (i.e., by interacting with host miRNAs) [27,28] may provide valuable insights into the conditions that promote or inhibit its activity and may help to identify new therapeutic targets. Second, delineating the molecular pathways through which viral proteins inhibit the antiviral functions of TRIM25 will allow researchers to identify potential therapeutic targets, thus paving the way for the development of antiviral agents that either enhance RIM25 activity or counteract viral evasion mechanisms. Third, there is currently no information on how TRIM25 binds to RNA viruses, independent of its known RNA-binding motif, given that this action may initiate viral mRNA degradation. In fact, TRIM25 overexpression is associated with decreases in viral replication. Hence, interference with TRIM25 expression is also a common strategy for viral evasion. Therefore, TRIM25 may serve as a suitable therapeutic target for enhancing antiviral responses to limit viral spread. Finally, future studies should consider the implication of TRIM25 in the context of emerging viral pathogens and the evolving landscape of viral immune evasion strategies. As new viruses emerge, understanding the role of TRIM25 in antiviral responses will be critical for developing effective vaccines and therapeutics.

## Figures and Tables

**Table 1:** The interaction between TRIM25 and viral components.

Viruses	Viral Proteins and/or RNAs	Interactions with TRIM25	References
SARS-CoV-2	NP	NPs interact with TRIM25 to prevent the binding of TRIM25 to RIG-I in order to inhibit RIG-I polyubiquitination and IFN production.	[70]
		SARS-CoV-2 NPs recruit TRIM25 and G3BP2 to enhance their interactions and inhibit subsequent RIG-I signaling.	[73]
		SARS-CoV-2 NPs inhibit the formation of SGs by interacting with G3BP1 to prevent the co-condensation of G3BP1 and TRIM25.	[72,74]
	NSP5	NSP5 inhibits the RLR-mediated IFN response by restricting antiviral SG formation.	[72]
	ORF6	ORF6 inhibits RIG-I activation and IFN production by decreasing the mRNA level of RIG-I and mediating the proteasomal degradation of TRIM25.	[65]
		ORF6 directly inhibits the nuclear translocation of IRF3/7 and STAT1 to induce IFN and ISG activity and trigger the IFN-mediated signaling pathway.	[65]
Influenza A viruses	RNA	TRIM25 inhibits IAV replication and assembly by binding to the positive strand of RNA, promoting RNA instability and degradation.	[75]
	NS1-A	NS1-A directly interacts with RIG-I to inactivate subsequent IFN transcription.	[76,77]
Influenza B viruses	NS1-B	NS1-B interacts with TRIM25 to relieve the inhibition of the NS1-B CTD region upon RIG-I ubiquitination, enhancing IFN and inflammatory cytokine production.	[78]
EBOV	vRNP	TRIM25 ubiquitinates EBOV vRNP NPs and exposes CpG-rich viral genome sequences for ZAP recognition and a subsequent antiviral response.	[79,80]
DENV-2	sfRNA	sfRNA binds to TRIM25 to prevent its deubiquitination, which prevents subsequent activation of RIG-I/IFN signaling.	[81]

## Data Availability

Not applicable.
